# Pre-symptomatic and asymptomatic transmission of COVID-19: Implications for control measures in Qatar

**DOI:** 10.5339/qmj.2021.59

**Published:** 2021-10-25

**Authors:** Abdelaziz M Tawe`ngi, Samantha Johnston, Soha Shawqi Albayat, Devendra Bansal, Shazia Ahmed, Mohamed A Sallam, Hamad Eid Al-Romaihi, Mohammed Al-Thani, Elmoubasher Farag, Mohamed M. Emara

**Affiliations:** ^1^Basic Medical Sciences Department, College of Medicine, QU Health, Qatar University, Doha, Qatar E-mail: eabdfarag@moph.gov.qa; ^2^Health Protection and Communicable Diseases, Public Health Department, Ministry of Public Health, Doha, Qatar

**Keywords:** COVID-19, pre-symptomatic transmission, public health control, Qatar

## Abstract

Public health control measures for communicable diseases are often based on the identification of symptomatic cases. However, emerging epidemiological evidence demonstrates the role of pre-symptomatic and asymptomatic transmissions of coronavirus disease 2019 (COVID-19). Understanding high-risk settings where transmissions can occur from infected individuals without symptoms has become critical for improving the response to the pandemic. In this review, we discussed the evidence on the transmission of severe acute respiratory syndrome coronavirus-2, its effect on control strategies, and lessons that can be applied in Qatar. Although Qatar has a small population, it has a distinct setting for COVID-19 control. It has a largely young population and is mostly composed of expatriates particularly from the Middle East and Asia that reside in Qatar for work. Further key considerations for Qatar and travel include population movement during extended religious holiday periods, screening and tracing of visitors and residents at entry points into the country, and expatriates living and working in high-density settings. We also consider how its international airport serves as a major transit destination for the region, as Qatar is expected to experience a rapid expansion of visitors while preparing to host the FIFA World Cup in 2022.

## Introduction

Traditional public health strategies that rely on the early detection of symptomatic cases were important for containing previous epidemics of severe acute respiratory syndrome (SARS) and Middle East respiratory syndrome (MERS); however, the coronavirus disease 2019 (COVID-19) has taken a different trajectory. The coronaviruses that cause SARS and MERS are associated with high case-fatality rates. In comparison, severe acute respiratory syndrome coronavirus-2 (SARS-CoV-2), the virus that causes COVID-19, is considered less fatal, but highly transmissible with more mild and asymptomatic infections^1^. SARS-CoV-2 is also associated with community spread rather than nosocomial spread^2^. Meanwhile, nosocomial outbreaks of MERS caused by MERS-CoV that occurred mainly in hospitals in Saudi Arabia, Jordan, and South Korea are considered to have limited transmissibility, even in the absence of control measures^3^. As the COVID-19 pandemic continues, it is important to consider how individuals without symptoms affect estimates of transmission, tracing and isolating of cases, and other control measures implemented in Qatar.

Qatar has a small population of 2.8 million and rapidly expanded its capacity in real-time polymerase chain reaction (RT-PCR) testing early in the pandemic^4,5^. As of 10 September 2021, 2,558,174 tests have been performed with 234,362 confirmed cases and 606 COVID-19-related deaths^6^. Qatar also has a distinct demographic profile with a young population, where only 1.5% are aged  ≥ 65 years^7^. Qatar further has a large expatriate population of 89% that comprise two main groups. First, over half of expatriates are young men, many of whom are craft and manual workers that generally live in high-density shared accommodation^8^. Second, other expatriates work in professional and service-related roles, and their accommodation tends to be separated into family and single households. As the country plans to host the International World Cup in 2022, a forecasted 1.7 million people may visit Qatar with approximately 500,000 visitors on its busiest days^9^. Given these settings, we discuss the current evidence on pre-symptomatic and asymptomatic transmission of COVID-19 and examples of how this has affected control strategies worldwide.

### Transmission Of Sars-cov-2

SARS-CoV-2 is predominantly transmitted person-to-person through respiratory droplets from coughing, sneezing, talking, and close contact. Indirect transmission or fomite transmission through contamination of surfaces by respiratory droplets has also been reported, particularly within healthcare facilities^10^ ^11^. Reported outbreaks in indoor settings such as gyms and restaurants with less ventilation suggest the possibility of aerosol transmission, where infectious droplets may remain in the air. However, it is difficult to establish whether droplet and fomite transmission can explain clusters in settings where measures such as hand hygiene, masks, and physical distancing are not maintained^12^. While traditional public health control measures often rely on the identification of individuals without symptoms, increased attention is also placed on transmission from individuals without symptoms. To better understand how, when, and what types of settings transmission occurs, it is important to also distinguish pre-symptomatic transmission from infected individuals before they develop symptoms and truly asymptomatic transmission from infected individuals who never develop symptoms^13^.

SARS-CoV-2 can be transmitted by individuals during the incubation period, during the time between exposure to the virus, and during the development of symptoms^14^. The estimated median incubation period of SARS-CoV-2 is 5–14 days^15^. The likelihood of pre-symptomatic transmission is further supported by studies on viral shedding that show it is highest around the onset of symptoms, followed by a progressive decline post-symptoms^16^. Several studies have demonstrated pre-symptomatic transmission within closed settings, including a United States nursing facility, where more than half of the residents who tested positive for COVID-19 were asymptomatic at the time^17^, and household clusters in China where individuals who tested positive were only exposed to pre-symptomatic family members^18,19^. Further mathematical modeling in Singapore and China has estimated that pre-symptomatic transmission contributed to 48% and 62% of secondary cases, respectively^20^. Another model in China estimated that 44% of secondary cases contracted the disease from an index case during the pre-symptomatic phase^21^. However, a study of 273 cases from seven clusters in Singapore reported that 6.4% of secondary cases were caused by pre-symptomatic transmission^22^. The reasons for the difference in modeling and observed cases remain unclear. In the absence of wide population-based antibody testing, the proportion of truly asymptomatic cases that never develop symptoms is difficult to estimate accurately and reports vary widely^23^. A meta-analysis has estimated the proportion to be 16%, ranging from 6% to 41%^24^. It further discussed limitations in available studies including unclear definitions of asymptomatic cases and incomplete follow-up of cases to determine if they remained truly asymptomatic^24^. Another model has estimated that up to 24% of SARS-COV-2 transmissions worldwide came from individuals without symptoms^25^. This is further supported by a study in China that identified and hospitalized 279 contacts of COVID-19 positive cases and found that 23% remained asymptomatic until discharge. The mean age of this cohort was 39 years, and 87% had no comorbidities^26^. In Qatar, an epidemiological study^4^ examined the first 5,685 COVID-19 cases from 28 February 2020 to 18 April 2020. The median age of the patients was 34 (IQR 28–43) years, of which 89% were male and 91% were non-Qatari expatriates. The majority (91%) had no symptoms or had mild symptoms, 2% had a severe or critical illness, and 84% of cases had no comorbidities. Although asymptotic and pre-symptomatic cases are not described, it does highlight that most cases occur among young, healthy individuals who only exhibit minimal symptoms and should be considered an important source of infection.

## Key Observations

### Control measures worldwide and implications for Qatar

As countries implement social and physical distancing, contact tracing, testing, and isolation with varying strategies, we discuss examples from similar settings and events in Qatar to guide current and long-term planning for COVID-19 control. Key priority areas include extended religious holiday periods, a high-capacity international airport, and expatriates living and working in high-density environments.

### Mass travel during holiday periods and travel restrictions

A significant challenge during the pandemic has been mass population movement for extended periods during major national holidays, which became evident for many countries early in the pandemic. China has the largest example, where mass migration occurred for the Lunar New Year Holiday and millions traveled for family visits within 40 days. Cases of atypical pneumonia were first reported in China's epidemic center Wuhan City, the capital of Hubei province, on 31 December 2019^27^. After travel restrictions were placed on Wuhan City on 23 January 2020, most Chinese cities had already received infected travelers^28^. One model predicted that this travel ban only resulted in modest delays of 3–5 days within mainland China^29^. Hence, the implementation and adoption of social and physical distancing policies became critical for limiting contact and preventing transmission from individuals who had traveled outside of Hubei province, especially from those without symptoms or showing mild symptoms. The Lunar New Year Holiday period was due to end on 31 January 2020; however, a key control measure by government authorities was to extend the dates. In effect, it postponed the return to work and schools, so that the duration of the holiday would sufficiently cover the suspected incubation period. Additionally, infected asymptomatic and pre-symptomatic individuals in Wuhan were quarantined in temporary field hospitals, while those outside of Wuhan were encouraged to quarantine at home until symptoms presented for treatment^30^.

Iran also experienced similar challenges of mass population travel for the Iranian New Year Holiday of Nowruz, as it became a new epicenter for COVID-19. Iran confirmed its first case of COVID-19, imported from China on 19 February 2020, which rapidly spread to most provinces before Nowruz commenced on 20 March 2020^31^. While restrictions were placed on international travel, domestic travel remained possible during the 2-week holiday period, and millions continued to visit family and friends and tourism locations across the country^32^. Within the first week of Nowruz, Iran experienced a subsequent peak in the number of cases^33^, and travel restrictions where citizens could not leave their cities of residence were subsequently placed on 11 April 2020 after the holiday period.

China’ and Iran's experiences highlight the importance of timing restrictions during peak periods of travel. Although Qatar has a much smaller population and size, the peak of the pandemic coincided with the 30-day period of Ramadan that commenced on 23 April 2020^6^. Ramadan is associated with large community gatherings, frequent visits to family and friends, and vacations following the last day of Eid on 23 May 2020. Mosques, large gatherings, and international travel were suspended during and after this time. The country also enforced further restrictions, including wearing of masks in public, restrictions on public and household social gatherings and on the number of passengers in vehicles. The first phase of a planned lifting of restrictions commenced weeks after Eid on 15 June 2020^34^, where limited mosques and commercial businesses with low capacity began reopening. A model has estimated that social and physical distancing interventions reduced peaks for incidence, prevalence, acute-care hospitalization, and intensive care unit hospitalization by 87%, 86%, 76%, and 78%, respectively^35^. As the pandemic progressed, Qatar continued to operate with a certain level of restrictions that were increased again in preparation for Eid the following year, with gradual lifting in three phases commencing after the holiday period on 28 May 2021^34^. Accordingly, well-timed and public communication of plans and periods for increasing and lifting restrictions has remained an important tool in the country.

### International airports and screening of travelers

In addition to domestic and international travel restrictions, border control must look for effective strategies for screening travelers. Current evidence indicates that temperature screening alone at entry and exit points is ineffective at preventing the entry of infected travelers. Thermal screening cannot detect infected individuals who are not showing symptoms, and fever can be temporarily reduced using antipyretics. One model estimated that 46% of infected travelers would not be detected at entry or exit points, even under best-case assumptions for the sensitivity of screening, incubation period, and proportion of infected travelers without symptoms^36^. Another model estimated that screening will miss more than half of infected individuals under best-case assumptions^37^. Temperature screening also requires a substantial cost investment for its limited effectiveness.

Thus, early in the pandemic, testing for SARS-CoV-2 was a critical tool to screen travelers returning to Qatar. Qatar identified the first cases of COVID-19 among quarantined returning travelers on 28 February 2020, which was followed by its first cluster of 300 cases by 6 March 2020. This prompted investment and rapid expansion of the testing capacity to approximately 4,000 tests per day performed by a central laboratory at Hamad Medical Corporation, the national public health service provider^4^. To further facilitate testing, important efforts included inviting individuals who had recently returned from overseas to a drive-through testing clinic, commencing on 2 March 2020. This enabled individuals to remain isolated in their vehicles and minimize transmission from congregating in a hospital's waiting area^38^.

A further challenge is supporting the exchange of health information between individuals and public health authorities. Qatar has utilized a mobile application known as Ehteraz as a contact tracing tool that further records an individual's status for testing and infection (positive, negative, or suspected), quarantine, and vaccination^39^. As of 10 September 2021, the application remains in routine use, and a negative infection status is required to enter indoor facilities, as well as a complete vaccination status to dine indoors and participate in other activities as part of government preventative measures^34^. Moreover, Qatar's Hamad International Airport is a major transportation hub between Europe, Asia, and the Middle East that served nearly 39 million passengers in 2019. Regarding the national airline, Qatar Airways was also the largest airline to continue services during the pandemic^40^. The airport's demand is also expected to increase to 53 million passengers annually by 2022 because Qatar will host the FIFA World Cup^9^. Methods that improve contact tracing and the collection of health information such as vaccination status, testing status, and travel history will be imperative in the long term as Qatar prepares for an increase in international visitors, in addition to supporting global travel by its citizens and expatriate residents.

### High-density settings

High-density work and household settings can increase the risk of transmission between individuals during the COVID-19 pandemic, and key examples are healthcare facilities, aged care facilities, cruise ships, and other residential facilities with shared and congregated housing. COVID-19 outbreaks across these settings have shown that when symptomatic cases are identified, asymptomatic cases are also frequently present^41^. The municipality of Vo’ in Italy's Veneto region provides an example of how mass community-based testing combined with case isolation, including those without symptoms, and community lockdowns can control local outbreaks. On 21 February 2020, Italy reported its first COVID-19-related death in the municipality of Vo’. Regional authorities promptly placed Vo’ in lockdown for 14 days and commenced testing of its 3,000 residents with isolation of all infected individuals, of which 43% were found to have no symptoms^42^. This early response resulted in a reduction of infected individuals from 2.6% to 0.3%, with no further cases of transmission as the remaining cases were effectively isolated. This wider testing strategy was adopted across the wider Veneto region. By contrast, the Lombardy region had twice the population of Veneto and only tested symptomatic individuals at this time. The number of tests per capita performed in Veneto was two times as high as Lombardy, while the case-fatality rate in Veneto was two times lower than that in Lombardy^43^. Accordingly, extending testing to include asymptomatic cases and close contacts has been increasingly recommended for the detection of asymptomatic cases, as well as understanding the local epidemiology of COVID-19. This must also consider the resources and capacity of health systems and testing laboratories to respond.

Within Qatar, several studies have tried to further understand the prevalence and risk of infection among select populations. Regarding demography, Qatar is often categorized as having a 40% professional population that consists of single and family households largely in office and service work versus a 60% population of craft and migrant workers^8^. A recent seroprevalence study in Qatar tested 112,941 individuals for SARS-COV-2 antibodies between May and September 2020, which indicates prior infection as opposed to PCR testing that detects the present infection. It found that less than 20% of the professional population was positive for antibodies. Of those positive, 47% had received a prior PCR-confirmed diagnosis^44^. By contrast, a separate seroprevalence study tested 2,641 craft and manual workers between July and September 2020, of which 55% had SARS-COV-2 antibodies, while 11% were PCR-positive^45^. The higher prevalence of infection among craft workers was associated with occupation, with higher odds found among security, transport, cleaning, technical, and construction workers than among professional workers. Craft and migrant workers are also considered more vulnerable to infection due to shared high-density accommodation, transportation, and equipment^5,45^. These results also suggest that the country's restrictions were more effective at reducing infection among its professional population, but they remained vulnerable to future outbreaks, while craft and manual workers were closer to reaching herd immunity^44^. In an additional study on surveillance data in Qatar on 201,006 individuals between March and July 2020, those employed in the private sector had a higher positivity rate than those employed in the government sector^46^. Wider testing reveals gaps in protection across the population. However, since the time of these studies, Qatar has continued its vaccination strategy, and as of 12 September 2021, over 78% of the total population aged  ≥ 16 years are fully vaccinated with two doses of Pfizer or Moderna^6^.

A further study conducted extensive testing of 16,912 healthcare workers employed at Hamad Medical Corporation, which includes 12 hospitals, the National Ambulance Services, and tertiary care services^47^. Of these, 11% were COVID-19 cases confirmed by RT-PCR and 67% reported experiencing at least one symptom. The study revealed several important considerations for control of transmission within healthcare settings: the vast majority (95%) had acquired infection at a non-COVID-19 facility, many reported exposure to a colleague (45%) or patient (29%) at a non-COVID-19 facility, and adherence to full protective personal clothing was significantly less in non-COVID-19 facilities (68%) than in dedicated COVID-19 facilities (82%). As restrictions are lifted in line with the decline of detected COVID-19 cases and efforts continue to prevent a resurgence in cases, sustained access to testing particularly in high-density and high-risk settings will be a key measure to contain community outbreaks.

## Conclusion

Factors that apply to Qatar that can increase the risk of transmission include family and social gatherings during religious holidays, a high-capacity international airport, high-density accommodation, and work environments. Our ability to predict periods of high transmission, use of methods to improve contact tracing and sharing of information, and implementation of a broad testing strategy to reduce the risk of transmission are imperative, not only in the current situation but in the long term as the country prepares to receive a substantial increase in the number of visitors during the FIFA World Cup in 2022 and protect its citizens and residents.
